# Treatment outcomes of patients with FIGO Stage I/II uterine cervical cancer treated with definitive radiotherapy: a multi-institutional retrospective research study

**DOI:** 10.1093/jrr/rrv036

**Published:** 2015-06-24

**Authors:** Takuro Ariga, Takafumi Toita, Shingo Kato, Tomoko Kazumoto, Masaki Kubozono, Sunao Tokumaru, Hidehiro Eto, Tetsuo Nishimura, Yuzuru Niibe, Kensei Nakata, Yuko Kaneyasu, Takeshi Nonoshita, Takashi Uno, Tatsuya Ohno, Hiromitsu Iwata, Yoko Harima, Hitoshi Wada, Kenji Yoshida, Hiromichi Gomi, Hodaka Numasaki, Teruki Teshima, Shogo Yamada, Takashi Nakano

**Affiliations:** 1 Department of Radiology, Graduate School of Medical Science, University of the Ryukyus, 207 Uehara, Nishihara-cho, Okinawa, 903-0215, Japan; 2 Research Center for Charged Particle Therapy, National Institute of Radiological Sciences, Chiba, Japan; 3 Department of Radiation Oncology, Saitama Cancer Center, Saitama, Japan; 4 Department of Radiation Oncology, Tohoku University School of Medicine, Sendai, Japan; 5 Department of Radiology, Saga University, Saga, Japan; 6 Department of Radiology, Kurume University Hospital, Fukuoka, Japan; 7 Division of Radiation Oncology, Shizuoka Cancer Center Hospital, Shizuoka, Japan; 8 Department of Radiology and Radiation Oncology, Kitasato University School of Medicine, Kanagawa, Japan; 9 Department of Radiology, Sapporo Medical University, Sapporo, Japan; 10 Department of Radiation Oncology, Hiroshima University Graduate School of Biomedical & Health Sciences, Hiroshima, Japan; 11 Department of Clinical Radiology, Graduate School of Medical Sciences, Kyushu University, Fukuoka, Japan; 12 Department of Diagnostic Radiology and Radiation Oncology, Chiba University Graduate School of Medicine, Chiba, Japan; 13 Gunma University Heavy Ion Medical Center, Gunma University, Gunma, Japan; 14 Department of Radiation Oncology, Nagoya Proton Therapy Center, Nagoya City West Medical Center, Aichi, Japan; 15 Department of Radiology, Takii Hospital, Kansai Medical University, Osaka, Japan; 16 Department of Radiation Oncology, Miyagi Cancer Center, Miyagi, Japan; 17 Division of Radiation Oncology, Kobe University Graduate School of Medicine, Hyogo, Japan; 18 Department of Radiation Oncology, St Marianna University, School of Medicine, Kanagawa, Japan; 19 Department of Radiation Oncology, Osaka Medical Center for Cancer and Cardiovascular Disease, Osaka, Japan; 20 Department of Radiation Oncology, Gunma University Graduate School of Medicine, Gunma, Japan; 21 Department of Radiation Oncology, National Hospital Organization, Fukuyama Medical Center, Hiroshima, Japan; 22 Department of Medical Physics and Engineering, Osaka University Graduate School of Medicine, Osaka, Japan

**Keywords:** cervical cancer, early stage, radiotherapy

## Abstract

The purpose of this study was to analyze the patterns of care and outcomes of patients with FIGO Stage I/II cervical cancer who underwent definitive radiotherapy (RT) at multiple Japanese institutions. The Japanese Radiation Oncology Study Group (JROSG) performed a questionnaire-based survey of their cervical cancer patients who were treated with definitive RT between January 2000 and December 2005. A total of 667 patients were entered in this study. Although half of the patients were considered suitable for definitive RT based on the clinical features of the tumor, about one-third of the patients were prescribed RT instead of surgery because of poor medical status. The RT schedule most frequently utilized was whole-pelvic field irradiation (WP) of 30 Gy/15 fractions followed by WP with midline block of 20 Gy/10 fractions, and high-dose-rate intracavitary brachytherapy (HDR-ICBT) of 24 Gy/4 fractions prescribed at point A. Chemotherapy was administered to 306 patients (46%). The most frequent regimen contained cisplatin (CDDP). The median follow-up time for all patients was 65 months (range, 2–135 months). The 5-year overall survival (OS), pelvic control (PC) and disease-free survival (DFS) rates for all patients were 78%, 90% and 69%, respectively. Tumor diameter and nodal status were significant prognostic indicators for OS, PC and DFS. Chemotherapy has potential for improving the OS and DFS of patients with bulky tumors, but not for non-bulky tumors. This study found that definitive RT for patients with Stage I/II cervical cancer achieved good survival outcomes.

## INTRODUCTION

Several retrospective studies have reported favorable outcomes for patients with cervical cancer who were treated with definitive radiotherapy (RT), not only for early-stage, but also for advanced-stage cancer [1–9]. One randomized clinical trial (RCT) found that there was no significant difference in the overall survival (OS) of patients treated with surgery and those treated with definitive RT [10]. After the results of that RCT, the clinical practice guidelines of the National Comprehensive Cancer Network (NCCN) recommended both surgery and definitive RT as treatment modalities for patients with resectable early-stage uterine cervical cancer [11, 12]. The RCT also found significantly poorer outcomes for patients with bulky tumor (diameter >4 cm) who underwent either surgery or definitive RT [10]. Therefore, additional treatment is thought necessary for patients with bulky tumors. Although several RCTs of neoadjuvant chemotherapy (NAC) followed by surgery versus surgery alone have been performed, none demonstrated improved survival for the NAC arm [13, 14]. Intermediate or high-risk pathological findings in the surgical specimen are indications for adjuvant treatments such as postoperative RT or concurrent chemoradiotherapy (CCRT) [11, 12]. However, increased incidence and grades of complications were reported for patients treated with surgery followed by postoperative RT [10]. On the other hand, several RCTs have demonstrated that definitive CCRT improved survival compared with RT alone [15]. The effect was significant, especially for patients with FIGO Stage I or II uterine cervical cancer [15]. Based on these findings, it seems reasonable to choose definitive RT or CCRT as the first treatment, except for some surgical cases who would not need adjuvant RT/CCRT.

The Japan Society of Obstetrics and Gynecology (JSOG) have periodically conducted a nationwide clinical practice pattern survey of uterine cervical cancer. Although the Japan Society of Gynecologic Oncology (JSGO) guidelines have recommended either surgery or definitive RT as treatments for early-stage cervical cancer [14], the JSOG survey reported that only 7% of patients with Stage I cervical cancer and 33% of patients with Stage II disease were treated with RT or CCRT [16].

Most clinical data on RT for Stage I/II Japanese cervical cancer patients have been derived from the experience of single institutions with small numbers of patients. Additional evidence on the efficacy and safety of RT for patients with early-stage cervical cancer is needed before the use of RT for these patients will increase. In addition, there is no available information on the use of RT for patients with bulky disease, although treatment results from a prospective multicenter study of RT for non-bulky disease have recently been reported [17]. The objective of this retrospective study was to analyze the treatment outcomes of a large number of patients with early cervical cancer who were treated with RT at multiple Japanese institutions.

## MATERIALS AND METHODS

The Japanese Radiation Oncology Study Group (JROSG) sent a questionnaire-based survey to 18 institutions that treated patients with FIGO Stage I/II uterine cervical cancer between January 2000 and December 2005 using definitive RT. Data were sent back to the data center at the Department of Medical Physics and Engineering, Osaka University.

The study was approved by the institutional ethical committee affiliated with the study chair (University of the Ryukyus). The questionnaire consisted of the following items: age, FIGO stage, indications for RT, pathology, maximum tumor diameter, lymph node status, modalities used for evaluation, start and end date of external beam radiotherapy (EBRT), total dose and dose per fraction of EBRT (with or without midline block), dose rate of intracavitary brachytherapy (ICBT), dose prescribing point of ICBT, total dose and dose/fraction (fr) of ICBT, chemotherapy regimen and timing of delivery (concurrent or not), starting date of chemotherapy, date of recurrence, recurrence site, and date and site of adverse effects (rectum, small intestine, bladder, other organs). The median follow-up time of all patients was 65 months (range, 2–135 months).

The Kaplan–Meier method was used to derive estimates of the OS, pelvic control (PC), and disease-free survival (DFS) rate. For all tests, *P* values < 0.05 were considered statistically significant. The tests for equivalence of the estimates of OS, PC and DFS consisted of the Breslow and log-rank tests. Multivariate analysis was performed using the Cox proportional hazards regression model. Adverse effects that occurred 90 days or more from the start of treatment were defined as late complications. Late complications were classified according to the Radiation Therapy Oncology Group (RTOG) late morbidity scoring criteria [18].

## RESULTS

A total of 667 patients were entered in this study. Patients treated with ICBT alone were excluded. Table 1 shows the number of patients from each institution. Table 2 summarizes the characteristics of the patients, focusing on the tumor. Although half of the patients were considered suitable for definitive RT, based on the features of the tumor, about one-third of the patients were prescribed RT instead of surgery because of age or poor physical condition.

**Table 1.** Participating institutions

Institution	Number of patients
National Institute of Radiological Sciences	114
University of the Ryukyus	100
Saitama Cancer Center	72
Tohoku University	58
Saga University	52
Kurume University	45
Shizuoka Cancer Center Hospital	34
Kitasato University	31
Sapporo Medical University	27
Hiroshima University	25
Kyushu University	23
Chiba University	22
Gunma University	18
Nagoya City University	17
Kansai Medical University	10
Yamagata University	9
Kobe University	7
St Marianna University	3
Total:	667

**Table 2.** Patient and tumor characteristics (*n* = 667)

Characteristics	*n*	%
Median age (year): 63 (range: 24–95)		
FIGO stage		
IA	1	
IB	199	30
IB1	122	18
IB2	51	8
IB unknown	26	4
IIA	87	13
IIB	380	57
Pathology		
SqCC	612	92
Adeno + AS	46	7
other	9	1
Primary tumor diameter		
median (mm): 41 (range: 3–125)		
<4 cm	262	39
≥4 cm	352	53
unmeasurable	53	8
	667	
Lymph node metastasis^a^		
negative	512	78
positive	145	22
unknown	10	1
Indication for definitive radiotherapy:		
Characteristics of the cancer	334	50
Unsuitable for surgery (e.g. poor physical condition)	239	36
Patient's decision	47	7
Other	47	7

^a^Lymph nodes ≥10 mm in minimum diameter by computed tomography or magnetic resonance imaging. SqCC = squamous cell carcinoma, Adeno = adenocarcinoma, AS = ademosquamous carcinoma.

Table 3 summarizes the details of RT and chemotherapy. The median total dose of EBRT without a midline block (MB) was 30 Gy/15 fr (range, 0–65 Gy), and the median total dose with MB was 20 Gy/10 fr (range, 0–50.4 Gy). The median total dose of ICBT at point A was 24 Gy/4 fr (range, 5–35 Gy for high-dose-rate [HDR] and 20–54 Gy for low-dose-rate [LDR]). The median overall treatment time for RT was 47 days (range, 14–160). The most frequent chemotherapy was concurrent delivery of cisplatin (CDDP).

**Table 3.** Details of radiotherapy and chemotherapy

	*n*	%
Radiotherapy (*n* = 667)		
EBRT		
Whole pelvic field	622	94
Extended field	27	4
Small pelvic field^a^	10	1
Others or details not available	8	1
	667	
ICBT		
HDR-ICBT	637	95
LDR-ICBT	24	4
No ICBT	6	1
Chemotherapy (*n* = 306)		
Concurrent	268	88
Neoadjuvant	18	6
Adjuvant	2	
Intra-arterial injection	5	2
Details were not available	13	4

^a^Small pelvic field excluded the common iliac region. EBRT = external beam radiotherapy, ICBT = intracavitary brachytherapy, HDR = high-dose-rate, LDR = low-dose-rate.

**Fig. 1.**
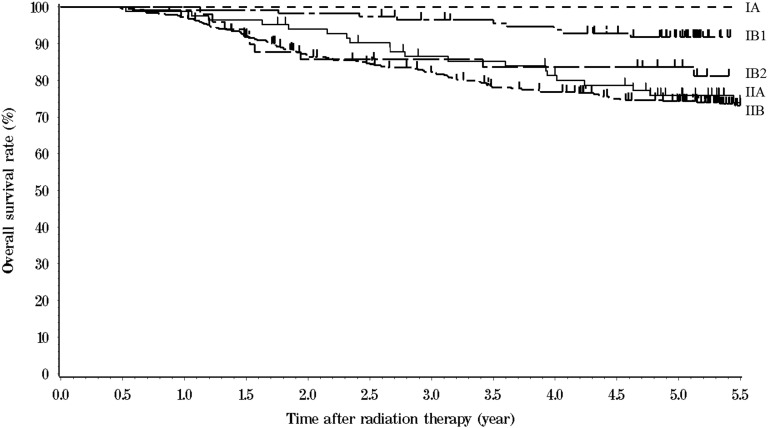
Overall survival curves of cervical cancer patients treated with definitive radiotherapy according to FIGO stage.

The five-year OS, PC and DFS rates for all 667 patients were 78% (95% confidence interval [CI], 75%–81%), 90% (95% CI, 88%–93%) and 69% (95% CI, 66%–73%), respectively. Mortality included 113 patients who died of cervical cancer, and 45 patients who died of other causes. Figure 1 shows the OS curves according to FIGO stage. Table 4 shows the 5-year actuarial outcomes of various tumor-related factors. Patients with adenocarcinoma and adenosquamous carcinoma had significantly poorer OS and DFS than patients with squamous cell carcinoma, but there was no significant difference in PC. Patients with bulky tumor (≥4 cm) had significantly poorer OS, PC and DFS than those with non-bulky tumors. Patients with lymph node metastasis had significantly poorer OS, PC and DFS than those without nodal metastasis. Table 5 shows the 5-year actuarial outcomes according to maximum tumor size and lymph node status.

**Table 4.** Five-year actuarial outcomes according to tumor-related factors (*n* = 667)

		(*n*)	OS (%)	PC (%)	DFS (%)
FIGO stage					
	IA	1	100	100	100
	IB	199			
	IB1	122	92	98	86
	IB2	51	84	96	77
	IB unknown	26	65	91	61
	IIA	87	76	89	68
	IIB	380	74	88	65
			*P* < 0.001	*P* = 0.003	*P* = 0.001
Pathology	SqCC	612	80	91	72
	Adeno + AS	46	61	89	50
			*P* = 0.001	NS	*P* = 0.001
Maximum tumor diameter					
	<4 cm	262	83	93	75
	>4 cm	352	75	88	65
			*P* = 0.02	*P* = 0.01	*P* = 0.004
Lymph node metastasis					
	Negative	512	82	92	75
	Positive	145	65	83	50
			*P* < 0.001	*P* < 0.001	*P* < 0.001


OS = overall survival, PC = pelvic control, DFS = disease-free survival, SqCC = squamous cell carcinoma, Adeno = adenocarcinoma, AS = adenosquamous carcinoma.

**Table 5.** Five-year actuarial outcomes by tumor size/nodal status

		*n*	OS (%)	PC (%)	DFS (%)
Tumor size^a^	Nodal status				
Bulky	Positive^b^	119	64	82	49
Bulky	Negative	230	80	91	74
Non-bulky	Positive^b^	23	68	90	56
Non-bulky	Negative	237	84	94	77
			*P* < 0.001	*P* = 0.003	*P* < 0.0001

OS = overall survival, PC = pelvic control, DFS = disease-free survival, ^a^Bulky = maximum tumor diameter ≥4 cm. ^b^Lymph nodes with minimum diameter ≥10 mm as measured by computed tomography or magnetic resonance imaging.

For all 667 patients, no significant differences were observed in the respective OS, PC or DFS of patients treated with RT alone or those treated with chemoradiotherapy (5-year OS: 81% vs 76%, *P* = 0.14; 5-year PC: 89% vs 92%, *P* = 0.13; 5-year DFS: 81% vs 76%, *P* = 0.98).

Chemotherapy was administered to 68% of patients with bulky tumors (240 of 352) and 79% of lymph node metastasis patients (114 of 145). Chemotherapy was most frequently administered to these high-risk patients concurrently with RT.

Table 6 summarizes the 5-year actuarial outcomes as a function of tumor size/nodal status and treatment. Among the patients with bulky tumors, OS and DFS were significantly better for patients treated with chemotherapy than for those who did not undergo chemotherapy, and there was no significant difference in PC between the two treatment groups. For patients with non-bulky tumors, there were no significant differences in OS, PC or DFS between the patients who were treated with CCRT and those who received RT alone.

**Table 6.** Five-year actuarial outcomes as a function of tumor size/nodal status and treatment

		*n*	OS (%)	PC (%)	DFS (%)
Tumor size					
Bulky	RT	112	60	86	56
(≥4 cm)	CRT	240	81	88	70
			*P* = 0.002	NS	*P* = 0.02
Non-bulky	RT	206	83	94	76
(<4 cm)	CRT	56	81	90	72
			NS	NS	NS
Nodal status					
Positive^a^	RT	31	43	77	36
	CRT	114	71	85	54
			*P* = 0.062	NS	NS
Negative	RT	325	79	91	73
	CRT	187	87	93	80
			*P* = 0.016	NS	NS

OS = overall survival, PC = pelvic control, DFS = disease-free survival, RT = radiotherapy alone, CRT = chemoradiotherapy. ^a^Lymph nodes with minimum diameter ≥10 mm as measured by computed tomography or magnetic resonance imaging.

Among lymph-node–positive patients, OS was better for patients treated with chemotherapy than for those who did not undergo chemotherapy. There were no significant differences in the PC or DFS between the two treatment groups. For patients without lymph node metastasis, similar trends were observed. OS was better for patients treated with chemotherapy than for those who did not receive chemotherapy, and there were no significant differences in PC or DFS between the two treatment groups.

Table 7 summarizes the results of multivariate analysis of outcomes according to prognostic factors. Administration of chemotherapy had a significant impact on OS and DFS, but there was no significant impact on PC.

**Table 7.** Multivariate analyses for outcomes according to prognostic factors

	OS (%)			PC (%)			DFS (%)		
	HR	95%CI	*P*	HR	95% CI	*P*	HR	95% CI	*P*
Age (<63 vs ≥63)			NS			NS			NS
FIGO Stage (IB1 vs IB2 vs IIA vs IIB)	1.4	1.2–1.7	0.0005	2.0	1.3–2.9	0.001	1.3	1.1–1.5	0.0003
Pathology (SCC vs Adeno/AS)	2.3	1.3–4.3	0.005			NS	2.3	1.4–3.9	0.0009
Tumor diameter (<4 cm vs ≥4 cm)			NS			NS			NS
Lymph node status (negative vs positive)	2.1	1.4–3.1	0.0003	1.9	1.0–3.4	0.05	2.6	1.9–3.7	<0.0001
Administration of chemotherapy (no vs yes)	0.4	0.3–0.6	<0.0001			NS	0.6	0.4–0.8	0.0006

HR = hazards ratio, CI = confidence interval, OS = overall survival, PC = pelvic control, DFS = disease-free survival, Adeno = adenocarcinoma, AS = adenosquamous carcinoma.

Recurrence developed in 159 of 667 patients as follows: 60 patients (9%) had pelvic recurrence alone, 86 patients (13%) had distant metastases only, and 13 patients (2%) developed both pelvic recurrence and distant metastases. The most frequent site of distant metastasis was the extrapelvic lymph nodes. The rate and site of metastases to lymph nodes were as follows: 52 patients (8%) had para-aortic metastatic lymph nodes, 18 patients (3%) had scalene nodes, 12 patients (2%) had mediastinal nodes, and 4 patients (1%) had other nodes. Other sites of metastases were as follows: 35 patients (5%) had lung metastases, 16 patients (2%) had bone metastases, 5 patients (1%) had liver metastases, and 4 patients (0.6%) had brain metastases.

Late complications developed in 178 patients (27%). There were 35 patients (5%) who developed severe (Grade 3 or higher) complications. The 5-year severe complication rate was 5.5% (95% CI: 3.6%–7.9%). The details are shown in Table 8. LDR-ICBT had a significant impact on the incidence of severe complications (*P* = 0.036). Other factors (total dose of EBRT without MB, total dose and dose/fr HDR-ICBT, administration of chemotherapy) did not have a significant impact on the incidence of severe complications.

**Table 8.** Details of late complications* (*n* = 667)

	Grade 1		Grade 2		Grade 3		Grade 4		Total	
	*n*	%	*n*	%	*n*	%	*n*	%	*n*	%
Proctitis	63	9	30	4	5	1	2	0.3	100	15
Cystitis	14	2	20	3	3	0.4	3	0.4	40	6
Enterocolitis	12	2	17	3	8	1	2	0.3	39	6
Others	11	2	17	3	7	1	8	1	43	6

*Some patients had complications in multiple organs. Toxicity was judged by the Radiation Therapy Oncology Group late morbidity scoring criteria.

## DISCUSSION

To the best of our knowledge, this is the largest study (*n* = 667) of patients with early cervical cancer who were treated with definitive RT, mainly with HDR-ICBT. The standard Japanese RT schedule achieved favorable survival rates and acceptable rates of complications that were comparable with previous studies [1–9].

The JSOG survey reported that the 5-year OS rates of patients with Stage I cervical cancer who were treated with surgery or RT were 93% and 80%, respectively, and the rates of Stage II patients were 81% and 74%, respectively [16]. The 5-year OS rates of our Stage I/II patients treated with RT were similar to the results of the JSOG RT group, but poorer than the results of the JSOG surgery group. Landoni *et al*. reported the results of their RCT, showing that the 5-year OS of Stage IB–IIA patients undergoing surgery was 83%, which was equivalent to the survival of patients undergoing definitive RT [10]. Selection bias might partially account for why the results of our study were inferior to the JSOG surgery group. The JSOG survey reported that ≥90% of Stage I patients and 67% of Stage II patients were treated with surgery [16]. It may be that the JSOG RT patients were mainly those who were unsuitable for surgery (poor general physical condition, elderly, metastatic lymph nodes). Our study might have had a similar selection bias. About one-third of our patients were elderly and/or in poor physical condition; one-third of the patients eventually died of other diseases.

A prospective study of definitive RT for patients with Stage I/II cervical cancer without bulky tumor or lymph node metastasis demonstrated an excellent 3-year PC of 96% and a 3-year OS of 95% [17]. In our study, the patients without bulky tumors and lymph node metastasis achieved good OS compared with the JSOG patients who underwent surgery. Even though they were treated with RT alone, non-bulky tumor and/or node-negative patients achieved good OS, PC and DFS. In contrast, our study patients with bulky tumors and/or lymph node metastasis had poor OS and PC. RTOG9001, a RCT of definitive CCRT, also included patients with Stage I/II cervical cancer with bulky tumors (>5 cm) and/or lymph node metastasis, as well as Stage III/IVA patients [19]. The outcomes of RTOG9001 were good for patients with Stage I/II disease treated with CCRT; the 5-year PC was 87% and the 5-year OS was 79%.

Our study patients treated with CCRT achieved PC and OS, similar to RTOG9001. We believe the fact that our outcomes were inferior to those of the JSOG patients undergoing surgery might have been accounted for by the low rate of chemotherapy administration to our patients. In our study, only 70% of patients were treated with chemotherapy, while the remaining patients did not receive chemotherapy even if they had bulky tumors and/or lymph node metastasis. This lower rate of chemotherapy treatment for high-risk patients might have adversely affected our outcomes, which were slightly worse than the JSOG surgery patients. In our study, patients with bulky tumors who were treated with chemotherapy achieved significantly better OS and DFS compared with patients who did not receive chemotherapy, and the same trend was observed for the patients with metastatic lymph nodes. Our results indicate that chemotherapy did not provide additional improvement of local control for Stage I/II patients, although OS and DFS were improved by chemotherapy. Based on these findings, although chemotherapy acts as an RT sensitizer, we think that the predominant role of chemotherapy for Stage I/II patients is to prevent distant metastases. Patients with bulky tumors and/or lymph node metastasis have been regarded as being at some risk of distant micrometastasis; therefore, we believe that it is important to administer chemotherapy if applicable. Prospective clinical trials of CCRT for Stage I/II cervical cancer patients with bulky tumors and/or lymph node metastasis are warranted. Because it is believed that patients in poor physical condition or of advanced age may be poor candidates for chemotherapy, we should also conduct trials that investigate suitable regimens for elderly patients or those in poor physical condition. Mitsuhashi *et al.* reported acceptable toxicity for low-dose CDDP for elderly patients [20]. Several reports have shown that nedaplatin achieved good survival with acceptable toxicity [21, 22]. We think that conducting clinical trials using similar less toxic regimens or drugs is valuable.

However, our patients with non-bulky tumors achieved good OS, PC and DFS, even though they were treated with RT alone. These findings are consistent with the results of a prospective clinical trial previously performed in Japan [17]. Taken together with our results, the findings suggest that for patients with non-bulky tumors, RT alone may be an adequate treatment for achieving good PC, OS and DFS.

Our study had some limitations. Some patients in this study had inadequate follow-up periods. This could be a critical flaw. If an adequate follow-up had been achieved for all patients, the outcomes might have changed. The JSOG's annual survey for survival analysis only includes patients from institutions with follow-up rates of >80% for treated patients [16]. A national cancer registration system that can achieve an adequate follow-up should be developed. Another limitation was on the types of data collected in this study. Unfortunately, we minimized the numbers of survey items to reduce the workload of our collaborators.

In conclusion, this study demonstrated that definitive RT for patients with Stage I/II cervical cancer achieved excellent PC. The results indicate that definitive RT can be considered the treatment of choice for patients with early-stage cervical cancer. However, it was difficult to compare the survival outcomes of our series directly with the outcomes from surgical series because of the diversity of patient backgrounds in our series. Prospective studies of definitive CCRT for patients with bulky tumors and/or lymph node metastasis are warranted. Moreover, for appropriate outcome reports, we suggest that a national database of patients treated with RT in Japan should be developed.

## FUNDING

Funding to pay the Open Access publication charge for this article was provided by a Grant-in-Aid for Scientific Research A (No. 21249066) from the Ministry of Education, Culture, Sports, Science and Technology (MEXT); and the Japan Society for the Promotion of Sciences (No. 60158194); the National Cancer Center Research and Development Funds (23-A-21, 26-A-4 and 26-A-28) Japan.
