# Corrigendum to: Treatment outcomes of patients with FIGO Stage I/II uterine cervical cancer treated with definitive radiotherapy: a multi-institutional retrospective research study

**DOI:** 10.1093/jrr/rrv096

**Published:** 2016-01-08

Corrigendum to: Ariga T, Toita T, Kato S, et al. Treatment outcomes of patients with FIGO Stage I/II uterine cervical cancer treated with definitive radiotherapy: a multi-institutional retrospective research study. J Radiat Res 2015 Sep; 56(5): 841–8.

In the article “Treatment outcomes of patients with FIGO Stage I/II uterine cervical cancer treated with definitive radiotherapy: a multi-institutional retrospective research study.”, the affiliation of two authors, i.e. of Dr. Shingo Kato and Dr. Teruki Teshima were incomplete.

The full and correct affiliations are follows:

Takuro Ariga^1^, Takafumi Toita^1*^, Shingo Kato^2,21^, Tomoko Kazumoto^3^, Masaki Kubozono^4^, Sunao Tokumaru^5^, Hidehiro Eto^6^, Tetsuo Nishimura^7^, Yuzuru Niibe^8^, Kensei Nakata^9^, Yuko Kaneyasu^10,22^, Takeshi Nonoshita^11^, Takashi Uno^12^, Tatsuya Ohno^13^, Hiromitsu Iwata^14^, Yoko Harima^15^, Hitoshi Wada^16^, Kenji Yoshida^17^, Hiromichi Gomi^18^, Hodaka Numasaki^19^, Teruki Teshima^19,23^, Shogo Yamada^4^ and Takashi Nakano^20^

^1^Department of Radiology, Graduate School of Medical Science, University of the Ryukyus, 207 Uehara, Nishihara-cho, Okinawa, 903–0215, Japan

^2^Research Center for Charged Particle Therapy, National Institute of Radiological Sciences, Chiba, Japan

^3^Department of Radiation Oncology, Saitama Cancer Center, Saitama, Japan

^4^Department of Radiation Oncology, Tohoku University School of Medicine, Sendai, Japan

^5^Department of Radiology, Saga University, Saga, Japan

^6^Department of Radiology, Kurume University Hospital, Fukuoka, Japan

^7^Division of Radiation Oncology, Shizuoka Cancer Center Hospital, Shizuoka, Japan

^8^Department of Radiology and Radiation Oncology, Kitasato University School of Medicine, Kanagawa, Japan

^9^Department of Radiology, Sapporo Medical University, Sapporo, Japan

^10^Department of Radiation Oncology, Hiroshima University Graduate School of Biomedical & Health Sciences, Hiroshima, Japan

^11^Department of Clinical Radiology, Graduate School of Medical Sciences, Kyushu University, Fukuoka, Japan

^12^Department of Diagnostic Radiology and Radiation Oncology, Chiba University Graduate School of Medicine, Chiba, Japan

^13^Gunma University Heavy Ion Medical Center, Gunma University, Gunma, Japan

^14^Department of Radiation Oncology, Nagoya Proton Therapy Center, Nagoya City West Medical Center, Aichi, Japan

^15^Department of Radiology, Takii Hospital, Kansai Medical University, Osaka, Japan

^16^Department of Radiation Oncology, Miyagi Cancer Center, Miyagi, Japan

^17^Division of Radiation Oncology, Kobe University Graduate School of Medicine, Hyogo, Japan

^18^Department of Radiation Oncology, St Marianna University, School of Medicine, Kanagawa, Japan

^19^Department of Medical Physics and Engineering, Osaka University Graduate School of Medicine, Osaka, Japan

^20^Department of Radiation Oncology, Gunma University Graduate School of Medicine, Gunma, Japan

^21^Department of Radiation Oncology, Saitama Medical University International Medical Center, Saitama, Japan

^22^Department of Radiation Oncology, National Hospital Organization, Fukuyama Medical Center, Hiroshima, Japan

^23^ Department of Radiation Oncology, Osaka Medical Center for Cancer and Cardiovascular Disease, Osaka, Japan

*Corresponding author. Department of Radiology, Graduate School of Medical Science, University of the Ryukyus, 207 Uehara, Nishihara-cho

We sincerely apologize for this inconvenience.

